# The Catalytic Effect of a Mechanochemically Synthesized Co–Fe Metal–Organic Framework on the Thermal Decomposition Behavior of Ammonium Perchlorate–Aluminum Composite Mixtures

**DOI:** 10.3390/ma19122524

**Published:** 2026-06-11

**Authors:** Albina Abdrassilova, Lyazzat Mussapyrova, Aisulu Batkal, Irina Bagina, Oksana Chervyakova, Dinara Muktaly, Sanat Tolendiuly, Kaster Kamunur

**Affiliations:** 1 Institute of Combustion Problems, Almaty 050012, Kazakhstan; albinabdrs@gmail.com (A.A.); lyazzat.mussapyrova@gmail.com (L.M.); abatkalova@mail.ru (A.B.); dinara.muktaly@mail.ru (D.M.); sanat_tolendiuly@mail.ru (S.T.); 2 Faculty of Chemistry and Chemical Technology, Al-Farabi Kazakh National University, Almaty 050040, Kazakhstan; 3 LLP “AlmaDK”, Almaty 041605, Kazakhstan; bagina_irina@mail.ru (I.B.); oksana_almadk@mail.ru (O.C.)

**Keywords:** metal–organic framework (MOF), mechanochemical synthesis, thermal decomposition, kinetic analysis, activation energy

## Abstract

In this work, the catalytic effect of a mechanochemically synthesized Co–Fe metal–organic framework (Co–Fe-MOF) on the thermal decomposition behavior of composite ammonium perchlorate–aluminum (AP-Al) systems was studied. The structural and morphological properties of the synthesized catalyst were characterized by X-ray diffraction (XRD), Fourier transform infrared spectroscopy (FTIR), and scanning electron microscopy (SEM). The results confirmed the formation of a highly dispersed Co–Fe-MOF structure with a heterogeneous surface morphology and uniformly distributed active regions, as observed by SEM. The thermal decomposition behavior of the composites based on AP was studied using differential scanning calorimetry (DSC) at different heating rates. The addition of Co–Fe-MOF significantly affected the thermal decomposition process, moving the main exothermic decomposition step towards lower temperatures. At 5 wt.% of catalyst, the decomposition temperature decreased from 438–467 °C to 358–398 °C. The kinetic parameters were evaluated using the Kissinger and Ozawa–Flynn–Wall methods. The activation energy decreased from around 191–200 kJ·mol^−1^ for pure AP and 184–194 kJ·mol^−1^ for the AP-Al system to 95–109 kJ·mol^−1^ after the introduction of 5 wt.% of Co-Fe-MOF. The observed catalytic activity is associated with accelerated electron transfer processes involving the redox couples Co^3+^/Co^2+^ and Fe^3+^/Fe^2+^, which favor the decomposition of AP and the oxidation of aluminum. The results demonstrate that the mechanochemically synthesized Co–Fe-MOF is an effective catalyst to improve the thermokinetic performance of AP-based energetic systems.

## 1. Introduction

Solid propellants are among the most energy-intensive materials that are widely used in the aerospace and defense industries. Among such systems, ammonium perchlorate (AP)-based composite propellants have found widespread use as oxidizer–fuel systems [1,2,3]. In particular, AP–Al systems with aluminum addition exhibit high heat release and relatively stable combustion [4,5,6]. In addition, the combustion efficiency of such systems is not always high. This is primarily due to the limitations of the heat and mass transfer processes occurring during combustion, as well as the relatively slow course of the oxidation–reduction reactions between the oxidant and the metal fuel [7,8,9,10]. As a result, complete oxidation of aluminum may not be ensured, leading to a lower burning temperature and a lower overall energy yield of the system.

To solve these problems, the use of catalytic additives that accelerate combustion and increase the intensity of oxidation reactions is widely considered [11,12,13,14]. In this regard, in recent years, metal oxides [15,16,17], carbon materials [18], and various nanostructured compounds [19,20,21] have been studied to improve the thermal decomposition of AP-based systems. However, traditional catalysts have several limitations. Among them, a lack of active centers, a tendency of particles to agglomerate, and a decrease in stability at high temperatures are often observed. Such factors limit catalyst efficiency and complicate their application in energy systems.

In recent years, metal–organic frameworks (MOFs) have been widely studied as catalytic materials in the chemical industry and in scientific research [22,23,24]. Their heterogeneous surface morphology, accessible active centers and agreeable structural characteristics enable efficient utilization and heterogeneous catalysis [25,26]. Bimetallic MOF systems containing two different metallic ions are of particular interest because the interactions between them can contribute to an increase in catalytic activity [27,28]. The selection of Co and Fe as mixed-metal centers for the construction of the MOF catalyst was based on elevated redox activity and their synergistic catalytic behavior. Transition metal ions such as Co^3+^/Co^2+^ and Fe^3+^/Fe^2+^ can facilitate electron transfer reactions during the thermal decomposition of ammonium perchlorate. In addition, bimetallic Co–Fe systems can provide a synergistic effect, increase the density of active catalytic sites, and improve heat and mass transfer. The numerous properties of Co-Fe-MOF materials make them promising catalysts for AP-based energetic systems. It is known that MOF structures based on cobalt and iron ions exhibit high activity in redox processes, and in this regard, they are considered capable of accelerating the thermal decomposition of ammonium perchlorate [29]. However, the specific catalytic effect of such bimetallic systems, especially their influence on the thermokinetic parameters in composite systems based on AP-Al, has not been systematically studied. In addition, the synthesis of such materials by traditional solution methods typically involves several steps, is time-consuming, and is not always environmentally friendly [30,31]. In this regard, there has been increasing interest in mechanochemical synthesis methods recently [32]. This method allows you to avoid using solvents or significantly reduce their use, resulting in reduced energy consumption and synthesis time. At the same time, it is possible to control the structural parameters of the resulting materials to some extent. MOF structures obtained by mechanochemical methods exhibit high dispersion and a large number of active sites, which may enhance their catalytic properties. However, despite the growing interest in MOF-based catalysts for energy materials, the thermokinetic effects of mechanochemically synthesized bimetallic Co–Fe-MOF catalysts on AP-Al composite systems have not been systematically studied [33,34,35]. In particular, limited information is available on the influence of catalysts on the activation energy, thermal decomposition pathways, and reaction behavior of the AP-Al systems under different heating rates. Therefore, the novelty of this work lies in the solvent-free mechanochemical synthesis of a Co–Fe-MOF catalyst and in the in-depth study of its catalytic effect on the thermal decomposition kinetics of AP-Al composites. The effect of concentration on the thermal behavior and activation energy of the systems was quantitatively evaluated using the kinetic methods of Kissinger and Ozawa. In addition, the possible catalytic roles of the redox centers Co^3+^/Co^2+^ and Fe^3+^/Fe^2+^ in accelerating the decomposition process have been discussed.

In this work, the effect of a Co–Fe-MOF catalyst prepared by mechanochemical synthesis on the thermal decomposition kinetics of AP–Al-based composite systems was investigated. The structural and morphological features of the synthesized MOF materials were studied using various physicochemical methods. The thermal properties of the composite mixtures with different amounts of catalyst were analyzed by differential scanning calorimetry (DSC) at different heating rates. Based on the experimental results obtained, the kinetic parameters of the process were calculated using the Kissinger and Ozawa methods.

## 2. Materials and Methods

### 2.1. Initial Materials

In the study, ammonium perchlorate (NH_4_ClO_4_, ≥99%, analytical purity) was used as the oxidizing agent and aluminum powder (Al, ≥99%, mean particle size 10–50 micrometers) as the metallic fuel.

Iron(III) nitrate (Fe(NO_3_)_2_ · 9H_2_O) and cobalt(II) nitrate (Co(NO_3_)_2_ · 6H_2_O) were used to synthesize the Co–Fe-MOF catalyst. Sulfosalicylic acid (C_7_H_6_O_6_S) was used as an organic ligand. The carboxyl and sulfo functional groups in this compound enter into coordination interactions with metal ions, allowing for the formation of stable structures. Due to these properties, it was considered an effective component in the synthesis of metal–organic framework (MOF) materials. All reagents used in the work were of analytical purity and did not require additional purification steps. Ammonium perchlorate was dried at 80–100 °C before the experiment. This operation was aimed at limiting the influence of moisture in the starting material and improving the reliability of thermal results. The aluminum powder was stored under lock and key to prevent oxidation and aggregation. Before the mechanochemical synthesis, metal salts and organic ligands were measured out in the required stoichiometric ratios.

### 2.2. Mechanochemical Synthesis of the Co-Fe-MOF Catalyst

The Co–Fe-MOF catalyst was synthesized in the solid phase via mechanochemical synthesis. The starting reagents were iron(III) nitrate (Fe(NO_3_)_3_·9H_2_O) and cobalt(II) nitrate (Co(NO_3_)_2_·6H_2_O). Sulfosalicylic acid (C_7_H_6_O_6_S) was obtained as the organic ligand. The metal salts and ligands were measured in a previously calculated stoichiometric ratio (Fe:Co = 1:1). The synthesis was carried out in a planetary ball mill. The reaction mixture was placed in a steel drum, and mechanical processing was carried out in a ball mill with the following parameters: ball-to-powder mass ratio of 10:1, rotation speed of 400 rpm, and processing duration of 60 min. During grinding, an intense mechanical effect was observed in the system, creating conditions for the formation of coordination interactions between the metal ions and organic ligands. As a result, a bimetallic metal–organic structure containing cobalt and iron ions was formed. The obtained material exhibited high dispersity and uniform particle distribution. After the processing, the product was stabilized at room temperature. If necessary, the samples were gently dried at 80–100 °C. Finally, the composition, structure, and morphology of the resulting catalyst were investigated.

### 2.3. Analysis of the Structural, Phase and Morphological Properties of Mechanochemically Synthesized Catalysts Using Physicochemical Methods

Several physicochemical methods were used to determine the structural and morphological characteristics of the synthesized Co–Fe-MOF catalyst. To determine the catalyst’s chemical structure and functional groups, infrared spectroscopy was performed. Measurements were performed on a BRUKER ALPHA II instrument (Bruker Optics GmbH, Ettlingen, Germany), and the spectra were recorded in the range of 400–4000 cm^−1^. The results showed that a metal–organic structure formed upon interaction of the carboxyl and sulfo groups of sulfosalicylic acid with metal ions. The phase composition and crystal structure of the material were analyzed by X-ray diffraction. The analysis was carried out on a Drawell DW-XRD-27 mini diffractometer (Drawell, Shanghai, China). As a result of processing the diffractograms, the presence of crystalline and amorphous phases in the sample, as well as the degree of formation of the MOF structure, was assessed. The morphology and dimensional characteristics of the catalyst particles were studied using scanning electron microscopy. The studies were carried out on a Quanta 200i 3D (FEI, Hillsboro, OR, USA) microscope. This method allowed us to determine the uniformity of particle distribution, the level of aggregation, and the surface structure. Thermogravimetric analysis (TGA) of the synthesized Co–Fe-MOF was carried out using a BAXIT (BXT-TGA-103, Beijing, China) thermal analyzer under a N_2_ atmosphere at a heating rate of 10 °C·min^−1^ in the temperature range of 25–1200 °C to evaluate its thermal stability.

### 2.4. Preparation of Composite Mixtures

Condensed composite mixtures were prepared from ammonium perchlorate, aluminum powder, and a Co–Fe-MOF catalyst obtained by mechanochemical synthesis. The mass ratio of the main components was set at 75:25 (AP:Al). To evaluate the effect of the catalyst, the amount of Co–Fe-MOF was varied from 0 to 5 wt.%. Accordingly, the composition of the systems was varied in the following ranges: AP—70–75 wt.%, Al—20–25 wt.%, Co–Fe-MOF—0–5 wt.%. The system without the catalyst was considered a comparative (control) sample. Before preparing the samples, ammonium perchlorate was pre-dried and sieved to a 100–200 μm fraction. All components were accurately weighed in the required amount and mixed in a dry state. To achieve a uniform distribution of composite mixtures, the mixing process was carried out in a ball mill at about 150 rpm for 10 min. Such processing conditions ensure a homogeneous distribution of components, limit structural changes in aluminum parts to a minimum, and reduce the risk of pre-reaction of the mixture. The obtained samples were stored in sealed containers to avoid the influence of environmental moisture and used in their original form during the study of thermal characteristics.

### 2.5. Study of Thermal Properties of Composite Mixtures

The thermal behavior of composite mixtures based on AP-Al was studied by differential scanning calorimetry. The analysis was carried out on a DSC 600 instrument (PerkinElmer Inc., Waltham, MA, USA), and measurements were carried out in an inert gas environment; the samples were placed in aluminum crucibles. The heating process was carried out in the temperature range of 25–500 °C, at rates of 5, 10, 15 and 20 °C min^−1^. The obtained data were used for subsequent kinetic analysis. During the processing of DSC curves, the temperatures of onset (T_onset_), maximum (T_max_) and end (T_offset_) of exothermic processes, as well as thermal effects, were determined. These indicators allow us to characterize the thermal stability and decomposition characteristics of the studied systems. Based on the results obtained at different heating rates, the activation energy and a possible reaction mechanism were estimated. In addition, the effect of the Co–Fe-MOF catalyst was considered separately, and the shift in the exothermic peaks to lower temperatures and the change in reaction intensity were analyzed.

### 2.6. Methods of Kinetic Analysis of Composite Mixtures

The kinetics of the thermal decomposition of AP-Al-based composite mixtures were investigated using multi-rate analysis based on the DSC results. The study utilized data obtained at heating rates of 5, 10, 15, and 20 °C·min^−1^. The results at different rates served as the basis for determining the reaction’s activation energy and analyzing its progress.

The Kissinger method was used to determine the activation energy (Ea) [36]. This method is based on the temperatures (T_max_) corresponding to the maximum of the reaction rate and is expressed by the following equation:

(1) $$\ln\;\left(\frac{\beta }{T_{max}^{2}}\right)=\;\ln\;\left(\frac{AR}{E_{a}}\right)-\;\frac{E_{a}}{{RT}_{max}}$$

where β is the heating rate (°C · min^−1^), T_max_ is the maximum reaction temperature (°C), A is the preliminary exponential multiplier, R is the Universal Gas Constant (8.314 kJ·mol^−1^·k^−1^), and Eₐ is the activation energy (kJ·mol^−1^).

The slope of the ln(β/$T_{max}^{2}$) − 1/T_max_ dependence is equal to −E_a_/R.

For the kinetic calculations, the Tmax values corresponding to the main high-temperature exothermic decomposition stage were selected. This exothermic peak was considered because it represents the dominant energy-release process associated with the thermal decomposition of ammonium perchlorate and the subsequent oxidation of aluminum in the composite systems.

In addition, the isoconversion method—the Ozawa–Flynn–Wall method (OFW method)—was used to investigate the reaction kinetics in more detail [37]. This method is based on data obtained at different degrees of conversion (α) and is expressed by the following equation:

(2) $$\ln(\beta )\;=\;\ln\left(\frac{{AE}_{a}}{Rg(\alpha )}\right)-\;5.331\;-\;1.052\;\frac{E_{a}}{RT}$$

where β is the heating rate, T is the absolute temperature (°C), and g(α) is a function depending on the degree of conversion.

The activation energy is determined by the slope of the ln(β) − 1/T dependence − 1.052 · Ea/R.

In order to increase the reliability of the experimental results, each measurement was repeated at least three times. Based on the obtained data, the average values were calculated, and the scatter of the results was estimated within ±2–5%. During the DSC analysis, the main parameters were checked by comparing the results of several independent measurements. Linear approximation was performed for the dependencies identified during the kinetic analysis and the corresponding correlation coefficients (R^2^) were calculated, which allowed us to assess the consistency of the results obtained. Experimental data processing and graphical analysis were carried out using special software.

## 3. Results and Discussion

### 3.1. Study of Compositional, Structural and Morphological Characteristics of Co–Fe-MOF Structures Synthesized by Mechanochemical Method

**Figure 1:**
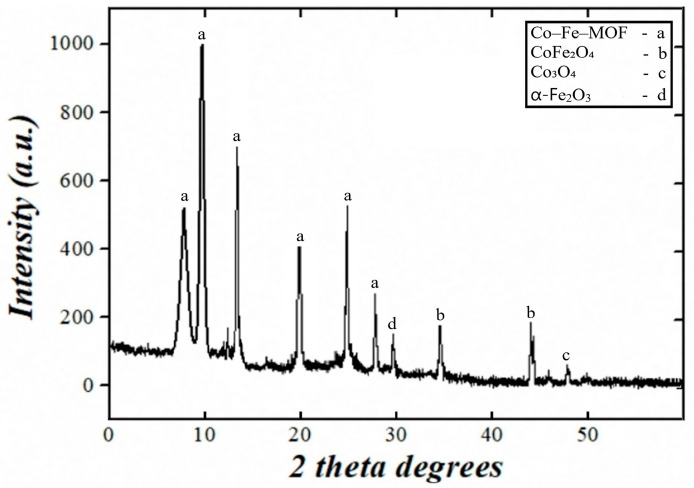
Results of X-ray phase analysis of Co–Fe-MOF material synthesized by mechanochemical method.

To evaluate the properties of the Co–Fe-MOF materials obtained by the mechanochemical method, their compositional features, structural organization and morphological characteristics were comprehensively studied. It is known that the structural organization, and surface morphology of MOF-type materials can significantly influence their catalytic and adsorption behavior [22,38]. In this work, the chemical composition, functional groups, crystal structure and particle morphology of the synthesized Co–Fe-MOF sample were determined using modern physicochemical analysis methods. Based on the results, the material’s structural particle distribution and level of aggregation were considered, and its application potential was assessed. It should be noted that the present work is primarily focused on evaluating the catalytic effect of the Co–Fe-based material synthesized mechanochemically in the AP-Al systems, rather than on the complete crystallographic resolution of a new MOF structure. Due to the solvent-free mechanochemical synthesis route, the obtained material exhibits highly dispersed and partially amorphous/polycrystalline characteristics, which limit the possibility of determining a single-crystal structure. Therefore, the structural identification of the synthesized material was carried out using complementary DRX, IRTF and SEM analyses. The diffraction peaks observed in Figure 1 are generally consistent with previously reported Fe-based MOF structures [39,40], indicating the formation of a metal–organic framework phase after mechanochemical synthesis. The phase attribution was based on the combined interpretation of the DRX, IRTF, and SEM analyses, as well as comparisons with previously reported diffraction data for MOFs containing Fe–Co and associated oxide phases.

**Figure 2:**
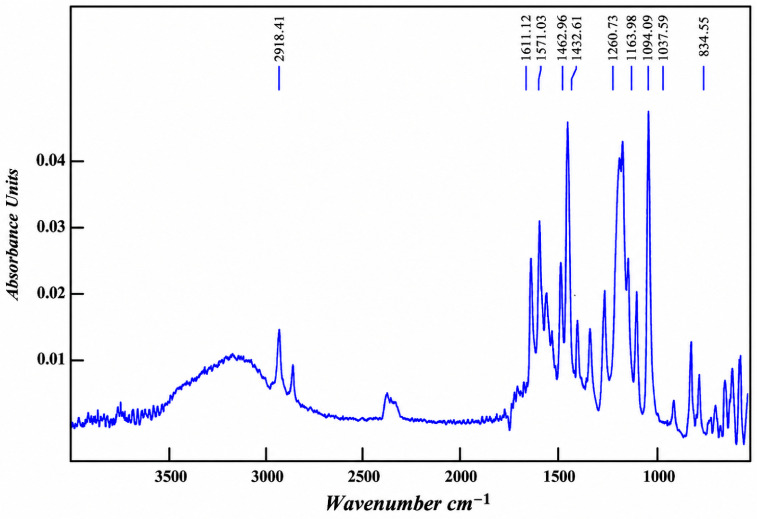
Infrared (IR) spectrum of Co–Fe–MOF material synthesized by mechanochemical method.

**Figure 3:**
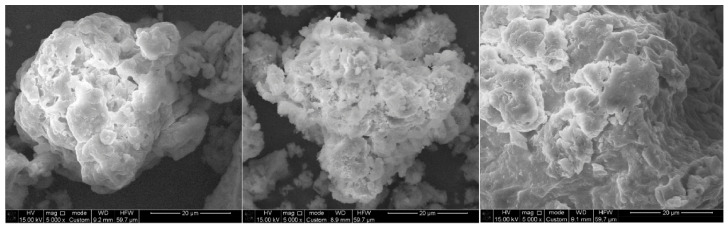
Morphology of mechanochemically synthesized Co–Fe-MOF.

The phase composition, structure, and morphology of the mechanochemically synthesized MOF material are shown in Figure 1, Figure 2 and Figure 3.

The results of the X-ray structure analysis shown in Figure 1 indicate that the main phase of the investigated sample is Co–Fe-MOF. High-intensity reflexes observed in the low-angle region indicate the formation of a crystal structure characteristic of metal–organic frameworks and are generally consistent with previously reported diffraction data for Fe-based MOF systems [39,40]. These results, together with FTIR and SEM analyses, support the formation of a Co–Fe-based metal–organic framework after mechanochemical synthesis. In addition, the registration of weak signals at large angles suggests the presence of additional phases in the sample. These reflexes, potentially, may correspond to oxide compounds such as CoFe_2_O_4_, Co_2_o_4_ and α-Fe_2_O_3_ [41]. The formation of these phases is considered the result of local high-energy effects and partial oxidation phenomena arising during mechanochemical processing [42]. However, the relatively weak intensity of these reflections indicates that the oxide phases are not present in minor quantities relative to the predominant Co–Fe-MOF structure. Therefore, the synthesized material was considered a composite system, mainly composed of Co–Fe-MOF, with minor oxidized phases. The individual catalytic contribution of each phase oxide has not been evaluated separately in the present study. Therefore, the catalytic behavior discussed in this work corresponds to the overall effect of the mechanochemically synthesized Co–Fe-MOF composite system. A detailed investigation of the individual catalytic role of CoFe_2_O_4_, Co_3_O_4_, and α-Fe_2_O_3_ phases will be carried out in future studies. Due to the overlap of some diffraction maxima, the specified phases were considered pre-matched. In general, it is observed that the Co–Fe–MOF structure predominates in the results obtained, and the oxide phases coexist in small quantities. According to the literature, in such multiphase systems, the combination of metal–organic structures and metal oxides can increase the material’s catalytic activity [43].

Figure 2 shows the infrared spectrum of the mechanochemically synthesized Co–Fe-MOF material. The spectrum reveals functional groups and the formation of metal–ligand coordination. A broad band in the range of 3400–3200 cm^−1^ corresponds to the vibrations of OH groups or adsorbed water molecules. This phenomenon indicates the presence of hydroxyl groups or unbound water molecules on the material’s surface. In addition, a weak band observed in the area of about 2918 cm^−1^ is characteristic of oscillations of C–H bonds, indicating that individual fragments of the organic ligand are preserved in the structure. The bands observed in the range of 1600–1500 cm^−1^ are due to asymmetric vibrations of C=C bonds and carboxyl groups (–COO^−^) in the aromatic ring. These changes suggest coordination between the ligand and the metal ions. In the range of 1250–1000 cm^−1^, the intense bands correspond to C-O and C-O-M bonds (M=Fe, Co). This indicates the formation of coordination bridges between the metal centers and the organic ligand. Strong signals in the range of 1100–1000 cm^−1^ indicate the formation of a spatial lattice of the MOF structure. The weak bands observed in the area of low frequencies correspond to the vibrations of metal–oxygen bonds (Fe-O and Co-O), which testify to the formation of metal nodes in the structure. In general, the results indicate that the Co–Fe-MOF material has been successfully synthesized and that stable coordination bonds have formed in its structure.

Figure 3 shows the morphology of the Co–Fe–MOF material obtained by the mechanochemical method. The studied sample consists mainly of fused (sintered) particles with a size of about 20–60 microns. Such a structure is explained by secondary agglomeration processes induced by high-energy collisions during mechanochemical processing. The particles’ surfaces are rough and uneven, indicating their high dispersibility and the presence of structural defects. In addition, there is an accumulation of small grains and nanoscale elements on the surface of the particle, which indicates the formation of many crystallization centers (nuclei). The subsequent merging of these centers leads to the formation of complex microclusters. Such morphological features are characteristic of mechanochemical synthesis, as the continuous formation of new reaction surfaces during grinding accelerates the rate of processes in the solid phase. As a result, the material exhibits a rough and heterogeneous surface morphology with a high degree of dispersion, which may contribute to the formation of accessible active regions for catalytic processes. The absence of pronounced crystallographic facets in the particles’ shape indicates an amorphous or semi-crystalline nature, consistent with the lack of long-term thermal treatment during mechanochemical synthesis. In general, the obtained SEM results indicate that the Co–Fe-MOF material has an agglomerated, porous and highly dispersed structure, which creates favorable conditions for improving its catalytic and adsorption properties. Although SEM analysis confirmed the heterogeneous morphology and highly dispersed structure of the synthesized material, additional elemental characterization techniques such as SEM–EDX mapping or ICP analysis are required for more precise verification of the Co and Fe distribution within the composite system.

**Figure 4:**
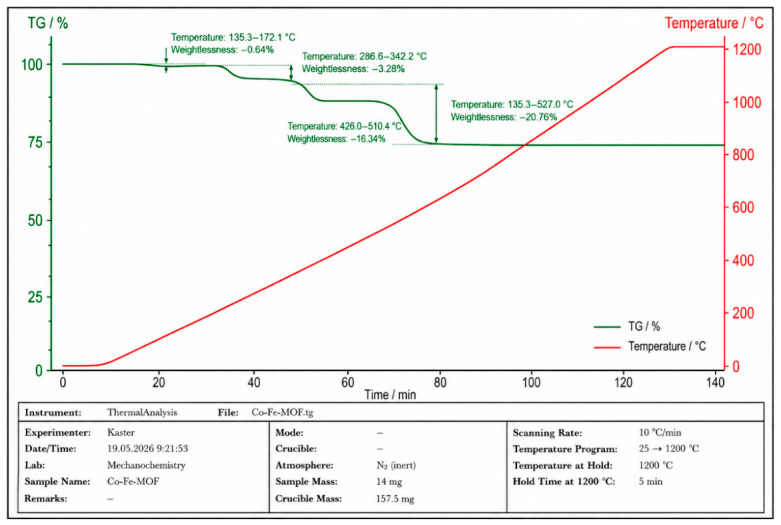
Thermogravimetric analysis (TG) curve of mechanochemically synthesized Co–Fe-MOF under N_2_ atmosphere.

To further evaluate the thermal stability of the mechanochemically synthesized Co–Fe-MOF material, thermogravimetric analysis (TGA) was carried out under an inert nitrogen atmosphere (Figure 4).

The TG curve demonstrated mass loss behavior in multiple stages. The minor initial weight loss of approximately 0.64% observed in the temperature range of 135.3 to 172.1 °C can be attributed to the elimination of physically adsorbed moisture and weakly bound species. The second stage is produced at 286.6–342.2 °C, with a weight loss of approximately 3.28%, and is associated with the initial decomposition of the organic ligand. The major decomposition stage was observed in the range of 426.0–510.4 °C with a mass loss of approximately 16.34%, corresponding to the degradation of the organic structure of the MOF structure. The total weight loss reached around 20.76%. At higher temperatures, the remaining mass remains relatively stable, which can be linked to the formation of phases containing thermally stable metals and oxide residues. These results indicate that although the partial decomposition of the organic framework occurs at elevated temperatures, the catalytically active centers containing Co and Fe remain present in the DSC temperature range studied and may contribute to the accumulated thermal decomposition of AP-based composites. Further structural investigations, including post-catalytic XRD analysis, are necessary to evaluate the structural stability of the composite after the catalytic decomposition process.

### 3.2. Results of the Thermal Analysis Experiment of the AP-Al and AP-Al-MOF Systems

**Figure 5:**
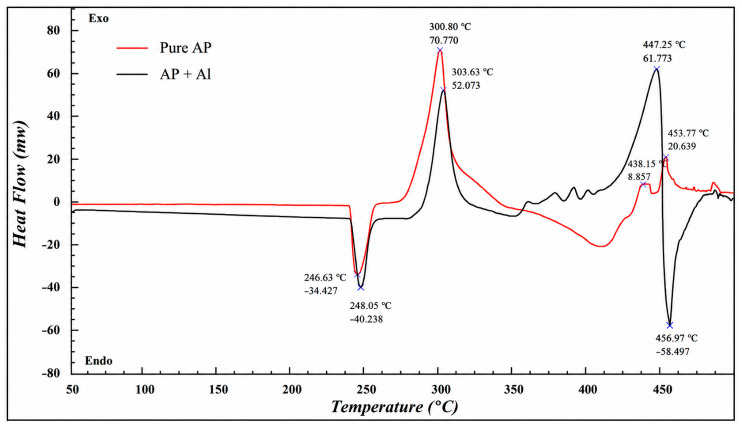
DSC curves of pure AP and AP-Al system.

**Figure 6:**
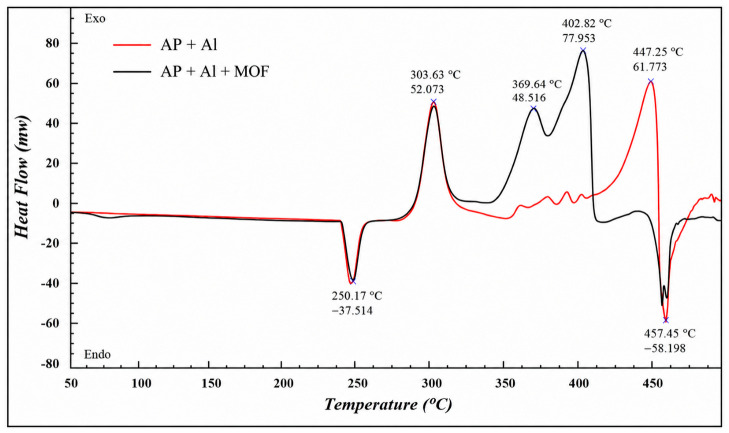
Comparative DSC curves of AP-Al and AP-Al/Co–Fe-MOF composite systems.

The study of the laws governing the thermal decomposition of AP-based composite systems is important for understanding the burning mechanism and the nature of energy release [2,3]. In particular, the introduction of aluminum powder and a MOF-type catalyst into the system significantly affects the reaction kinetics and thermal effects. As part of this study, the thermal characteristics of the AP, AP–Al and AP–Al–MOF systems were analyzed using DSC. The results obtained were analyzed comparatively (Figure 5 and Figure 6).

The DSC curves shown in Figure 5 are based on a comparison of the thermal decomposition mechanisms of pure ammonium perchlorate and the AP-Al system. For both systems, phase changes are observed at approximately the same temperature of 246–248 °C. At this stage, the crystal lattice is rebuilt, and the presence of aluminum in the system does not have a significant effect. However, the increased intensity of this effect in the AP-Al system indicates enhanced initial heat transfer processes. For pure AP, the main exothermic decomposition occurs at approximately 300.8 °C, and in this region the substance decomposes vigorously into gaseous products. In the AP-Al system, the temperature of the exothermic peak shifted slightly to a higher value (approximately 303.6 °C) under the influence of aluminum. At the same time, its intensity was relatively reduced. This change is due to early interaction of aluminum with the AP decomposition products, resulting in part of the released heat being spent on intermediate processes. The effect of aluminum on thermal decomposition is more pronounced high temperatures (≈400–460 °C). For pure AP, only a weak exothermic heat exchange process is recorded in this range. In the AP-Al system, a pronounced exothermic peak is observed at about 447 °C, indicating the active interaction of aluminum with oxidizing gases produced by the thermal decomposition of AP. In addition, the endothermic melting of aluminum is observed at about 457 °C. The melting process increases the reaction surface and enhances aluminum’s participation in gas-phase reactions, thereby affecting the intensity of subsequent exothermic reactions.

Figure 6 shows DSC curves for a comparison of the thermal decomposition characteristics of the AP-Al and AP-Al/Co–Fe-MOF systems. In both cases, the phase change process is observed at 250 °C, indicating that the MOF catalyst is independent of AP’s phase change. Differences are mainly observed in the exothermic region. In the AP-Al system, the main decomposition period occurs at about 303.6 °C. Although the position of this maximum does not change significantly in the system containing Co–Fe-MOF, the nature of the subsequent thermal processes is different. In particular, for the AP–Al/Co–Fe-MOF system, exothermic thermal decomposition occurs in two stages at about 369.6 °C and 402.8 °C. This is due to the effect of the MOF catalyst and indicates an acceleration of the further reaction of the decomposition products. This multi-stage process can be explained by the large surface area of the MOF structure and the catalytic activity of the Fe and Co centers. In addition, it is observed that oxidation of aluminum begins earlier and becomes more intense. This is confirmed by a high-intensity exothermic effect, observed at 402.8 °C. And in the AP-Al system, at a high temperature interval (about 440–460 °C), an exothermic maximum is observed near ≈447 °C, corresponding to the main oxidation stage of aluminum. In the presence of the MOF, this process becomes more complex and proceeds in several stages. The endothermic effect observed at approximately 457 °C is due to the melting of aluminum. As the melting increases, the reaction surface area of the aluminum intensifies the subsequent processes.

The study found that aluminum complicates the AP decomposition process, turning it into a multi-step reaction. The addition of a MOF catalyst increases the reaction rate, and exothermic reactions occur over a broader temperature range. This increases heat dissipation in the AP-Al/Co–Fe-MOF system and the overall reaction efficiency. According to the DSC results, MOF structures can serve as effective catalysts in ammonium perchlorate-based composite solid propellants. The results highlight the potential for using these materials to control the thermal and reaction properties of the condensed mixture. The AP decomposition kinetics were further investigated by calculating the activation energy (Ea).

### 3.3. Kinetic Parameters of the Ammonium Perchlorate–Aluminum and Ammonium Perchlorate–Aluminum–MOF Systems

**Figure 7:**
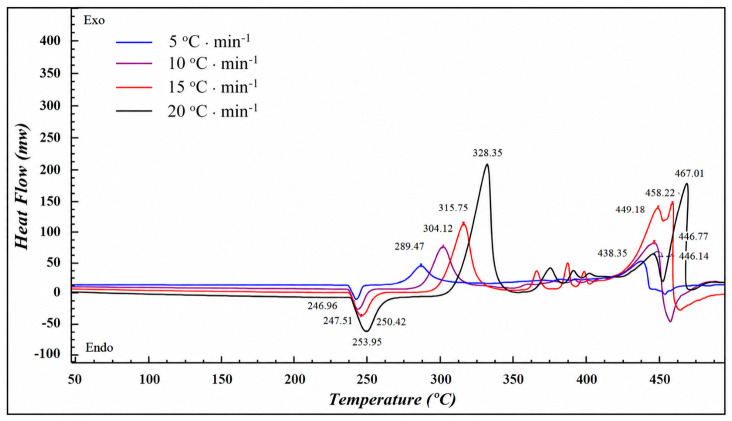
DSC curve of AP-Al composite mixture at different heating rates.

**Figure 8:**
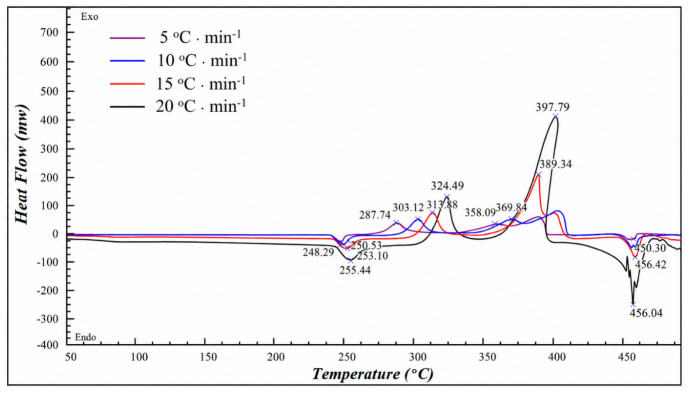
DSC curve of AP-Al composite mixture with MOF at different heating rates.

The combustion efficiency of solid rocket propellants is closely related to the kinetics of their thermal decomposition [1,3,6]. Therefore, it is important to determine the thermokinetic parameters for AP-based systems. In systems involving aluminum, the burning intensity largely depends on the reaction rate and the activation energy. The introduction of a MOF catalyst accelerates the decomposition process of ammonium perchlorate and changes its thermal behavior. In this work, the thermal characteristics of the AP-Al and Co–Fe-MOF systems were studied by differential scanning calorimetry at heating rates of 5, 10, 15 and 20 °C min^−1^. The results obtained are presented in Figure 7 and Figure 8.

The DSC curves presented here describe the characteristics of the thermal decomposition of AP-Al and AP-Al/Co–Fe-MOF systems at different heating rates (5–20 °C·min^−1^) and their kinetic behavior.

For the AP-Al system (Figure 7), an endothermic effect is observed in the DSC curves over ≈ 245–255 °C. This corresponds to the phase transition (orthorhombic → cubic) of ammonium perchlorate. This is followed by the main phase of exothermic decomposition in the range of 280–330 °C. Within the mentioned temperature interval, the thermal decomposition of ammonium perchlorate occurs, and the interaction with aluminum begins. As the heating rate increases, exothermic stages shift to higher temperatures, consistent with thermokinetic patterns. In the high temperature range (about 430–470 °C), additional exothermic effects are observed. These are related to the oxidation of aluminum and the intensification of gas-phase reactions.

In the second system (Figure 8), the thermal properties differ significantly. The endothermic phase transition period (≈248–255 °C) is retained, but the main exothermic peaks are shifted to the lower temperature range (≈287–324 °C). This phenomenon can be explained by the MOF catalyst’s effect. At the same time, it is observed that the intensity of exothermic maxima increases and their shape narrows. Such changes characterize the rapid and smooth course of the reaction. In the high temperature zone (about 360–400 °C), the main exothermic effect is enhanced and observed at a lower temperature than in the AP-Al system. This suggests that heat dissipation in the system has become more intense and the reaction mechanism has changed.

With an increasing heating rate, the temperature of the exothermic peaks in both systems increases. However, in the presence of the MOF, this shift is less pronounced, indicating a reduced activation barrier for the system. This phenomenon may be associated with the formation of active sites on the MOF surface and enhanced electron-transfer interactions between the oxidizing and combustible phases. Simultaneously, multi-stage decomposition is more pronounced in the presence of the MOF, meaning that intermediates are formed, which subsequently participate in exothermic reactions. The key thermal parameters determined by DSC analysis for the AP-Al-based systems are shown in Table 1.

The results presented in Table 1 show that the thermal parameters of the AP-Al and AP-Al/MOF systems depend on the heating rate. As the heating rate increases, the T_onset_, T_offset_ and T_max_ values for all samples shift to higher temperatures. This phenomenon may be associated with enhanced electron-transfer interactions at Co- and Fe-based active centers and increased reactivity at the interface of the oxidizing and combustible phases. This effect is especially pronounced in the second exothermic period, where a decrease in T_max_ is observed. A decrease in T_max_ in the second stage indicates a decrease in the reaction activation barrier in the presence of a catalyst. In addition, an increase in the amount of MOF leads to a decrease in thermal performance due to increased reaction activity and improved heat and mass transfer efficiency. This, in turn, increases the reaction rate and combustion efficiency. The results show that the catalytic effect of the MOF structures in the AP-Al system can be observed using temperature parameters.

**Figure 9:**
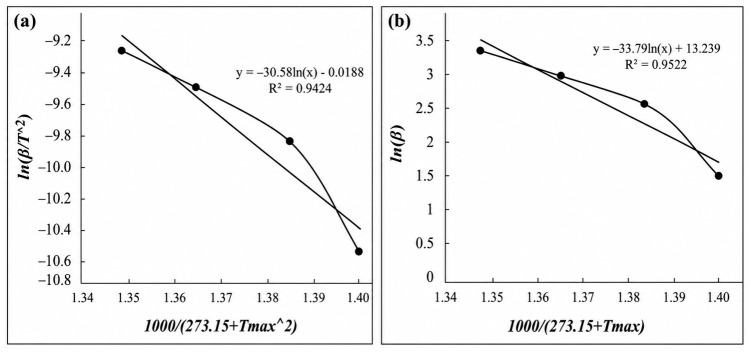
Kissinger (**a**) and Ozawa (**b**) plots of an AP-Al composite mixture.

**Figure 10:**
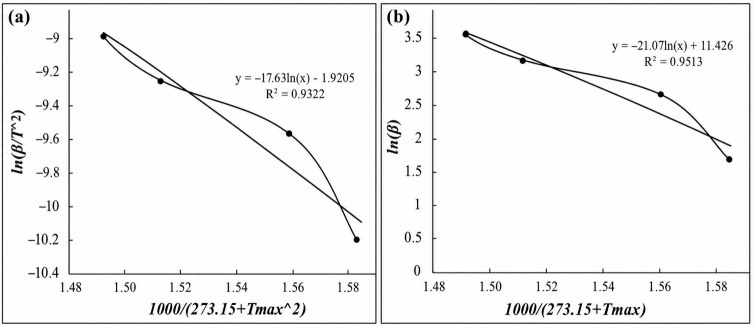
Kissinger (**a**) and Ozawa (**b**) plots of an AP-Al/Co–Fe-MOF composite mixture.

Changes in thermal parameters cannot fully describe the reaction mechanism. Therefore, it was necessary to quantify the kinetic parameters of the systems. For this purpose, the Kissinger and Ozawa methods were applied using the T_max_ values determined at different heating rates. These approaches allow for the determination of the activation energy and the comparison of catalyst activity. In the course of the calculations, the ln(β/T^2^) − 1/T and ln(β) − 1/T dependencies were constructed and the Kissinger and Ozawa graphs were obtained, respectively (Figure 9 and Figure 10). Based on the slope of the linear dependencies, the activation energy was determined and the effect of the MOF catalyst on the kinetic parameters was evaluated.

The Kissinger and Ozawa plots presented in Figure 9 and Figure 10 allow us to quantitatively characterize the thermal decomposition kinetics of the AP-Al and AP-Al/Co–Fe-MOF systems. The ln(β/T^2^) − 1/T and ln(β) − 1/T dependences obtained by both methods show a high linear agreement. The correlation coefficients obtained (R^2^ = 0.91–0.98) indicate good agreement between the applied experimental data and the kinetic models. The relatively high R^2^ values confirm the applicability of the Kissinger and Ozawa approaches to describe the thermokinetic behavior of the studied systems. At the same time, the slight differences observed across several samples can be associated with the decomposition mechanism and the several stages of composites based on AP, with exothermic processes that overlap and the formation of intermediate reaction products during thermal decomposition. Therefore, the variation in the values of R^2^ reflects the complexity of the decomposition process rather than the inadequacy of the kinetic models applied. The linear relationships obtained by the Kissinger and Ozawa methods indicate that these methods can be used for comparative kinetic evaluation of the studied systems. However, given the nature and number of stages of the thermal decomposition process and the presence of the MOF, the calculated activation energies should be considered apparent kinetic parameters.

The slope of a straight line in Kissinger’s graphs depends directly on the reaction’s activation energy, so its value characterizes the change in activation energy in the system. The dependencies determined the AP-Al system exhibit a relatively steep slope. This indicates that the reaction proceeds over a high activation energy barrier. In systems with a MOF, the slopes of the straight lines decrease, indicating that the catalyst reduces the reaction’s activation energy. A similar trend is also observed in the results obtained by the Ozawa method: the slope of the ln(β) − 1/T dependences decreases when a MOF is introduced, which indicates a decrease in the energy barrier of the reaction and a simplification of its kinetic characteristics. The agreement of the results obtained by the two methods confirms the correctness of the calculations and the reliability of the determined kinetic parameters. In addition, with an increase in the MOF amount, the curves’ slopes systematically decrease, indicating the effect of catalyst concentration. This phenomenon may be associated with enhanced electron-transfer interactions at Co- and Fe-based active centers and an increase in the reactivity at the interface of the oxidizing and combustible phases. As a result, the decomposition of ammonium perchlorate accelerates, and its temperature range of occurrence shifts to lower values.

The activation energies and correlation coefficients calculated from the graphical dependencies allow us to quantitatively characterize the kinetic parameters of the systems. These results are summarized in Table 2.

The kinetic parameters listed in Table 2 highlight the significant influence of the MOF catalyst on the thermal decomposition of the AP, AP-Al, and AP-Al-MOF systems. The high activation energy values for pure ammonium perchlorate (≈191–200 kJ mol^−1^) are explained by the stability of its crystal lattice and the multi-step decomposition mechanism. In the AP–Al system, this figure decreases slightly to about 184–194 kJ mol^−1^, due to a certain level of interaction between aluminum and the AP decomposition products, but the change is not significant. Upon introducing a MOF-type catalyst into the composition, a significant decrease in the activation energy is observed. At a catalyst content of 1 wt.%, Ea decreases to about 150–161 kJ mol^−1^, and at 3 wt.% it reaches the range of 133–145 kJ mol^−1^. The lowest values are recorded in the presence of 5 wt.% catalyst and are about 95–109 kJ mol^−1^, i.e., almost a two-fold decrease compared to the original system. These results indicate that MOF structures lower the reaction’s energy barrier and accelerate the thermal decomposition process. The decrease in activation energy can be associated with improved electron transfer processes at the active centers of Co and Fe, as suggested by the thermokinetic behavior and previously reported catalytic properties of Fe-Co-MOF systems. In general, the data obtained indicate that the Co-Fe-MOF catalyst acts as an effective additive in the AP-Al systems, significantly improving their thermokinetic properties. It is worth noting that the AP–Al/Co–Fe-MOF system exhibits thermal decomposition in several stages, particularly at higher catalyst contents. Therefore, the activation energy values calculated using the Kissinger and Ozawa methods represent approximate or apparent kinetic parameters corresponding to the dominant decomposition stages. More advanced or model-free kinetic approaches may provide a more comprehensive description of the reaction mechanism in future studies.

### 3.4. Proposed Reaction Mechanism of Co–Fe-MOF System

To substantiate the effect of the Co–Fe-MOF catalyst on the thermal decomposition of AP–Al-type composite systems, a possible reaction pathway was proposed. Ammonium perchlorate is characterized by the formation of gaseous products upon heating, which first undergo ionic decomposition. In this case, the ClO_4_^−^ anion overcomes a high energy barrier and forms intermediate species such as ClO_3_^−^, ClO_2_ and active oxygen radicals (O•) [2,3]. Without a catalyst, these transformations occur slowly and require high-temperature conditions. When a Co–Fe-MOF is introduced, the reaction rate in the system increases significantly. According to previously reported studies on Fe–Co-based MOF catalysts, the Fe^3+^/Fe^2+^ and Co^3+^/Co^2+^ redox couples are assumed to facilitate electron-transfer reactions during perchlorate decomposition [11,29]. In this regard, the metal–organic framework serves as an active center that facilitates electron transport in the reaction medium and accelerates the conversion of the initial anion into reactive intermediates. This phenomenon can be conditionally represented by a simple reaction diagram:

ClO_4_^−^ + e^−^ → ClO_3_^−^ + O• (3)

The oxygen radicals formed are highly reactive and interact strongly with aluminum particles. This contributes to the earlier onset of the aluminum oxidation process and can be demonstrated by the following reaction [6,8]:

Al + O• → AlO•(4)

In the presence of a MOF catalyst, the oxidation of aluminum begins at a relatively low temperature, thereby increasing the overall reaction rate. The heterogeneous morphology and dispersed structure of the Co–Fe-MOF-based material may facilitate interactions between reactive species and improve mass and heat transfer during thermal decomposition [22,26]. A portion of the gaseous products formed within the porous structure are temporarily retained, creating conditions for an increase in their local concentration. This, in turn, enhances the activation of radical chain reactions. In addition, active centers on the MOF surface accelerate radical formation during the reaction and contribute to the destruction of the oxide film on aluminum particles. As a result, the reactivity of aluminum increases, and the efficiency of the interaction of the oxidizing and combustible phases increases [6]. All this indicates a multi-stage nature of the thermal decomposition process in the AP–Al system, as evidenced by the appearance of additional exothermic stages.

The conducted research showed that the effect of the Co–Fe-MOF catalyst is realized through the combined effect of several interrelated factors. In particular, the participation of transition metal ions in redox processes, an increase in the intensity of the formation of active radicals, an increase in the reaction surface and improved mass transfer due to the heterogeneous surface morphology and highly dispersed structural features, as well as earlier onset of the aluminum oxidation process, play a key role. The combined effect of these factors reduces the reaction’s activation energy. As a result, the thermal decomposition process proceeds rapidly at relatively low temperatures. Nevertheless, direct experimental verification of the oxidation-state evolution by XPS or in situ spectroscopic methods is required for more detailed confirmation of the proposed mechanism and will be considered in future work.

## 4. Conclusions

The results of the study showed that the mechanochemically synthesized Co-Fe-MOF catalyst has a significant influence on the kinetics of the thermal decomposition of ammonium perchlorate–aluminum-based composite systems. Structural and morphological studies revealed that the obtained material has high dispersion, a clearly developed pore structure, and a uniform distribution of active metal centers. These features are correlated with its high catalytic activity. The results of differential scanning calorimetry showed that in systems with added Co–Fe-MOF, the thermal decomposition processes shift to lower temperatures. In particular, at a 5% concentration in the presence of a catalyst, the decrease in the temperature of the main decomposition reaction from 438–467 °C to 358–398 °C indicates an earlier reaction onset and increased reaction intensity. Furthermore, a multi-stage decomposition process with enhanced exothermicity was observed in the presence of the MOF. The results of the kinetic analysis highlighted a significant decrease in the activation energy of the reaction in the presence of the catalyst. The values of ≈191–200 kJ mol^−1^, typical of pure ammonium perchlorate, and ≈184–194 kJ mol^−1^ for the AP–Al system gradually decrease after the introduction of Co–Fe–MOF: at 1 wt.% to ≈150–161 kJ mol^−1^, at 3 wt.% to ≈133–145 kJ mol^−1^, and at 5 wt.% to ≈95–109 kJ mol^−1^. Such a change accelerates the thermal decomposition process of the catalyst and significantly reduces the energy barrier. According to the proposed mechanism, the variable oxidation states of Co and Fe ions (Fe^3+^/Fe^2+^ and Co^3+^/Co^2+^) facilitate electron-transfer interactions, accelerating the decomposition of the perchlorate anion and the formation of active oxygen species. In addition, the heterogeneous surface morphology and highly dispersed structural features inherent in the MOF structure increase the reaction area and increase the efficiency of mass and heat transfer processes. As a result, the degree of interaction between the oxidant and fuel components increases. In general, the Co–Fe–MOF catalyst obtained by the mechanochemical method is an effective additive to compositions based on AP–Al. However, the individual catalytic effects of the oxide phases present in the composite material were not individually investigated in the current study. Therefore, further studies are required to clarify the specific catalytic contribution of the CoFe_2_O_4_, Co_3_O_4_, and α-Fe_2_O_3_ phases to the overall thermal decomposition behavior. This study provides a significant opportunity to control the kinetics of thermal decomposition and increase the combustion efficiency of solid rocket fuels.

## Figures and Tables

**Table 1:** Thermal values of the DSC curve of AP-Al–based composite mixtures.

Sample	β (Heating Rate, °C · min^−1^)	Thermal Decomposition, °C					
		1st Step			2nd Step		
		T_onset_	T_offset_	T_max_	T_onset_	T_offset_	T_max_
0 wt.% MOF	5	276.32	341.12	289.47	416.07	451.55	438.35
	10	291.55	348.24	304.12	424.99	454.59	446.77
	15	302.01	354.39	315.75	431.56	461.98	458.22
	20	311.92	360.68	328.35	446.61	472.07	467.01
1 wt.% MOF	5	272.12	300.24	288.77	396.80	430.40	413.79
	10	286.58	313.15	303.72	412.91	445.21	424.04
	15	297.87	323.11	314.98	422.20	454.60	437.55
	20	307.27	332.77	327.12	428.81	461.07	446.25
3 wt.% MOF	5	269.54	296.21	288.15	365.21	398.71	380.01
	10	283.94	310.57	303.32	381.50	414.00	392.54
	15	294.11	320.41	314.31	390.91	423.51	403.21
	20	304.27	329.51	325.63	397.59	430.37	414.07
5 wt.% MOF	5	267.28	294.17	287.74	347.25	367.84	358.09
	10	281.74	309.05	303.12	360.21	379.78	369.64
	15	292.07	321.85	313.88	374.73	399.01	389.34
	20	302.25	329.33	324.49	390.07	407.23	397.79

**Table 2:** Kinetic parameters of AP-Al systems with and without MOF catalyst.

Composite Mixture	β (Heating Rate, °C · min^−1^)	T_max_, °C	E_a_/(kJ·mol^−1^) Kissinger’s Method	R^2^	E_a_/(kJ·mol^−1^) Ozawa’s Method	R^2^
AP	5	438.96	191.12	0.91	200.05	0.93
	10	447.61				
	15	455.21				
	20	467.44				
AP/Al	5	438.35	184.74	0.94	193.98	0.95
	10	446.77				
	15	458.22				
	20	467.01				
AP/Al/MOF (1 wt.%)	5	413.79	149.90	0.95	160.64	0.96
	10	424.04				
	15	437.55				
	20	446.25				
AP/Al/MOF (3 wt.%)	5	380.01	133.35	0.97	144.61	0.98
	10	392.54				
	15	403.21				
	20	414.07				
AP/Al/MOF (5 wt.%)	5	358.09	95.52	0.93	108.50	0.95
	10	369.64				
	15	389.34				
	20	397.79				

## Data Availability

The original contributions presented in this study are included in the article. Further inquiries can be directed to the corresponding author.
